# Osteonecrosis of the jaws in patients treated with bisphosphonates

**DOI:** 10.4317/jced.50649

**Published:** 2012-02-01

**Authors:** Fadi Ata-Ali, Javier Ata-Ali, Antonio J. Flichy-Fernández, José V. Bagan

**Affiliations:** 1 DDS. University of Valencia Medical and Dental School; 2 DDS. Primary care dentist, Valencian public healthcare service. Master in Oral Medicine and Surgery. Master in Oral Surgery and Implantology. Valencia University Medical and Dental School; 3 DDS. Master in Oral Surgery and Implantology. Valencia University Medical and Dental School; 4 Chairman of Oral Medicine. Valencia University Medical and Dental School. Head of the Department of Stomatology. Valencia University General Hospital. Valencia, Spain

## Abstract

The literature describes an increasing presence of bisphosphonate-induced osteonecrosis of the jaws (ONJ), characterized by the exposure for over 8 weeks of necrotic bone in the maxillofacial region, after bisphosphonate therapy, in the absence of prior maxillary radiotherapy. The present literature review examines the etiopathogenesis, risk factors, clinical forms, diagnosis, treatment and prevention of bisphosphonate-induced ONJ. In addition, a review is made of all the series involving over 15 patients diagnosed with this disorder between 1 January 2011 and 15 May 2011. A PubMed-Medline search was carried out with the following key words: “bisphosphonates” and “osteonecrosis”. The appearance of osteonecrosis is a serious complication, with an increasing incidence, that affects patient quality of life and causes important morbidity. All patients treated with bisphosphonates are at risk of developing osteonecrosis as a result of such medication. This potential complication therefore should be explained to the patient by both the prescribing physician and the dental surgeon in charge of oral treatment, with the obtainment of informed consent in all cases.

** Key words:**Osteonecrosis of the jaws, bisphosphonates, etiopathogenesis, prevention, treatment.

## Introduction

Bisphosphonate-induced osteonecrosis of the jaws (ONJ) was first described by Marx in the year 2003 (1). The disease is characterized by the exposure for over 8 weeks of necrotic bone in the maxillofacial region, after bisphosphonate (BP) therapy, in the absence of prior maxillary radiotherapy (2-4). Although the condition is typically confined to the maxillofacial region, there have been reports of cases in the hip, tibia and femur (5). The reason for such exclusive involvement of the jaws is subject to controversy. In this sense, many factors could be implicated, including the anatomical characteristics of alveolar bone, its fine overlying epithelial layer, mechanical stress caused by chewing, inflammatory processes (periodontitis), and a complex oral microbiota involving the presence of bacteria such as Fusobacterium, Bacillus, Actinomyces, Staphylococcus, Streptococcus, Selenomonas and Treponema (6,7). Oral bisphosphonates are indicated for the treatment of osteopenia, osteoporosis and Paget’s disease (4), while intravenous bisphosphonates are used in patients with cancer and bone metastases, for the prevention of bone complications (pathological fractures, spinal cord compression and problems related to bone irradiation and/or surgery), and for the treatment of tumor-induced hypercalcemia (4,8). The appearance of osteonecrosis is a serious complication that affects patient quality of life and causes important morbidity (2,9).

The incidence of ONJ is estimated to be 0.01-0.04% in the case of oral BP treatment, versus 0.7-12% in the case of intravenous BP administration (4,9) – this percentage reaching 21% when these drugs are administered for three or more years (10). In addition to the administration route employed, these differences are due to other factors such as the treatment indication, potency, administered dose and the duration of treatment (11).

The present study reviews the literature on the etiopathogenesis, risk factors, clinical forms, diagnosis, treatment and prevention of bisphosphonate-induced osteonecrosis of the jaws.

## Material and Methods

A PubMed-Medline search was carried out with the following key words: “bisphosphonates” and “osteonecrosis”. A total of 382 articles published in the last two years were reviewed. Of these, we excluded 31 studies in languages other than English and 30 studies in animals. Apart from these 321 initially considered articles, we also included other publications derived from manual searches and from references in review articles that were regarded as important. Study selection was based on a review of the titles and abstracts, with a view to obtaining the full texts of those publications considered to be of relevance in terms of the quality of their methodological design. The articles obtained from the search were classified as corresponding to the following areas: etiopathogenesis, risk factors, clinical forms and histology, diagnosis and treatment, and prevention. In addition,  Table 1 includes all the series involving over 15 patients diagnosed with bisphosphonate-induced ONJ between 1 January 2011 and 15 May 2011.

**Table 1:**
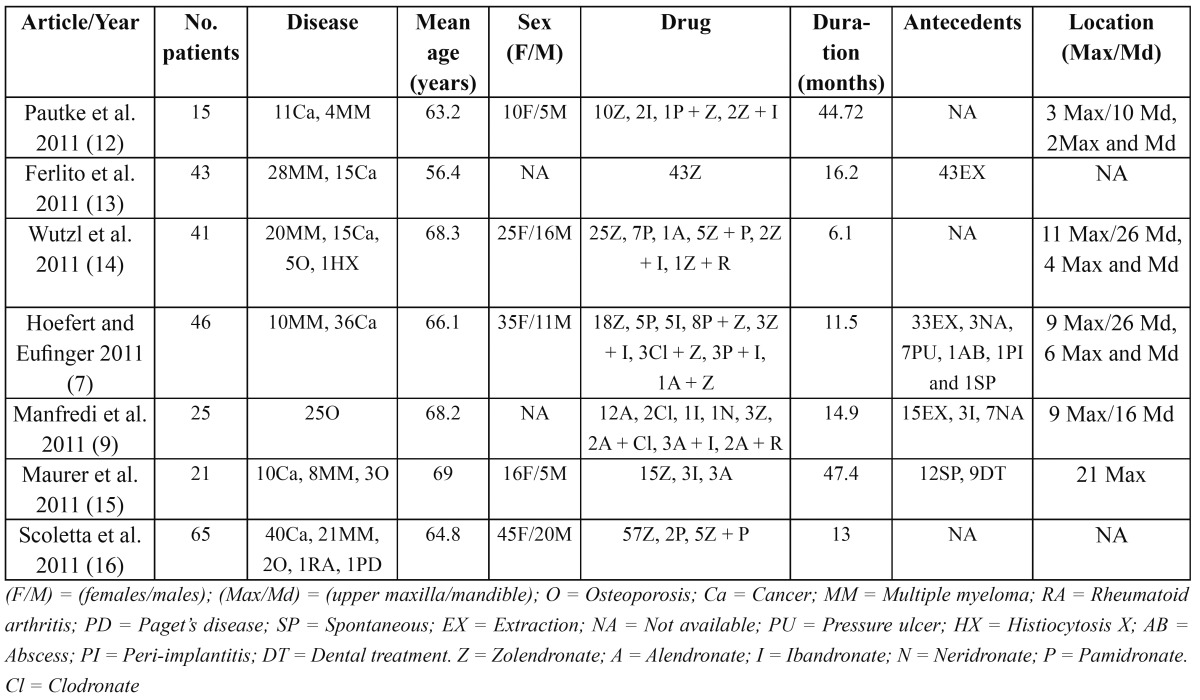
Bisphosphonate-induced osteonecrosis of the jaws in published series involving more than 15 patients.

## Etiopathogenesis

The molecular structure of the bisphosphonates is similar to that of the pyrophosphates, with the exception that the former have a P-C-P bond at the center of the structure (3). Bisphosphonates inhibit the enzyme 3-hydroxy-3-methylglutaryl coenzyme A reductase. Once bound to bone, they prevent bone resorption mediated by osteoclasts, and stimulate apoptosis of these cells. Bisphosphonates have also been reported to exert an antiangiogenic effect through the suppression of serum interleukin levels generated by the endothelial cells (3,17). A number of factors have been related to the etiopathogenesis of ONJ, such as immune disorders and alterations of the reparatory mechanisms, since 95% of all patients with ONJ present tumors as background disease. Although vascular impairment has been postulated as one of the key elements in the etiopathogenesis of ONJ, the latter has also been erroneously linked to avascular necrosis in other locations such as the hip, since there are no clinical or physiopathological parallelisms between the two disorders. Diminished bone turnover and toxicity at both bone level and in the soft tissues have also been cited as etiopathogenic factors (18). Bisphosphonates have been reported to act directly upon keratinocytes and fibroblasts, inhibiting their activities through aging processes and apoptosis. This in turn affects cell proliferation and migration, resulting in a lack of reepithelization of the oral mucosa (19). Although the physiopathology of ONJ remains to be fully clarified, the inhibition of bone remodeling has been suggested to play a significant role. The best evidence in support of this hypothesis comes from patients not treated with bisphosphonates. There have been reports of ONJ in patients treated with drugs such as denosumab that inhibit bone remodeling by acting upon the RANKL receptor (20). There have also been descriptions of ONJ without previous BP treatment in patients with herpes-zoster infections and HIV-positive individuals (21).

The half-life of the bisphosphonates in blood is short (between 30 minutes and 2 hours), though once bound to bone these drugs can persist within the body for years (1). Although the presence of bacteria has been demonstrated in patients with BP-induced ONJ, it is not clear whether infection is a primary or a secondary cause of the disorder (2,10).

Many patients present antecedents of local trauma, particularly dental extractions (70%), with a lesser incidence of other surgical procedures. ONJ has been reported to develop spontaneously in 30-50% of the cases (8,22), particularly in locations where the gingival mucosa is thinner (2,10).

In a review of 468 dental implants in 115 patients subjected to oral BP therapy, no cases of ONJ were observed, and only two implants failed. The success rate was therefore similar to that recorded in patients without BP treatment. In the absence of other diseases or medications, the placement of implants and their osteointegration during the first three years of treatment with oral bisphosphonates can be regarded as safe (23). In another retrospective study (24) involving implant placement in 61 patients treated with oral bisphosphonates for an average of 3.3 years, no cases of ONJ were recorded during follow-up (12-24 months), and the implant success rate was 100%. Nevertheless, it must be taken into account that a number of authors (7,9,11,22,25) have described cases of BP-induced ONJ in patients with dental implants.

## Risk factors

A number of studies (2-4,14,26) have analyzed the risk factors underlying of BP-induced ONJ. Treatment with potent intravenous bisphosphonates such as zolendronate or pamidronate, and tooth extractions, are the most important factors, with an estimated risk of between 6.7% and 9.1% after extraction (4). Other potentially influencing factors are periodontal or periapical surgical procedures, the presence of dental abscesses, anatomical factors such as the presence of a torus, the duration of BP treatment, the number of treatment cycles, diabetes, deficient oral hygiene and the concomitant administration of corticosteroids or thalidomide (2,6,17,27). A genetic influence has also been postulated in the development of the disease through the cytochrome P450-2C enzyme system (CYP2C8), since the latter is implicated in the arachidonic acid metabolism and cholesterol biosynthesis, and can modulate angiogenesis and osteoblast differentiation in bone (3,10).

## Clinical forms and histology

 Table 2 describes the different potential clinical stages of BP-induced ONJ. Patients complain of progressive and persistent pain, following an initially asymptomatic period. The disorder often produces suppuration through gingival fistulas, with posterior exposure of necrotic maxillary or mandibular bone through the mucosa. Clinically, these exposed bone areas measure between 0.5-2 cm in diameter, and several simultaneous exposure sites are frequently observed in one same patient (28). In addition, other less common manifestations include loss of sensitivity in the territory innervated by the inferior alveolar nerve (Vincent’s sign) (10). Regarding the location of these areas, mandibular involvement is much more common than maxillary bone exposure, and in the mandible the molar region is particularly susceptible (22) – possibly due to the presence of terminal vascularization (6) that cannot be compensated by peripheral perfusion from the gingiva and periodontal tissue.

**Table 2:**
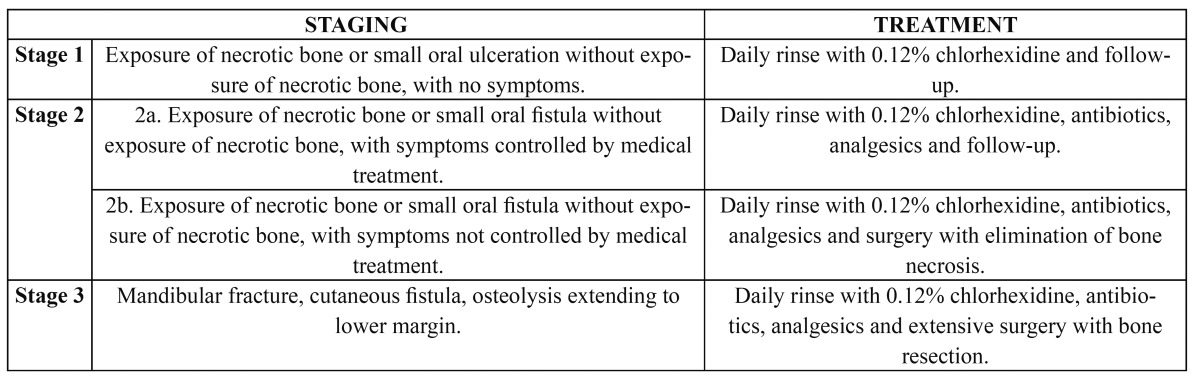
Proposed staging classification and treatment of bisphosphonate-induced osteonecrosis of the jaws (32)..

The histological characteristics in turn comprise osteomyelitis with an inflammatory infiltrate of the necrotic tissue, while the adjacent zones show the presence of Actinomyces and pseudoepitheliomatous hyperplasia (15).

## Diagnosis and treatment

A number of diagnostic criteria for osteonecrosis of the jaws have been proposed (8):

Patients receiving or who have received BP therapy.

The presence of one or more ulcerated lesions affecting the mucosa of the alveolar processes, with the exposure of maxillary or mandibular bone. There also may be cases without bone exposure, with pain or fistulas, that should be subjected to more detailed study.

Exposed bone of necrotic appearance.

Lesions manifesting spontaneously or, more often, after dental-alveolar surgery (particularly extractions).

Absence of healing over a period of at least 6 weeks.

Computed tomography (CT) is effective in assessing the size and extent of the necrotic bone, and magnetic resonance imaging (MRI) can also be used to evaluate osteonecrosis, showing diminished signal intensity in T1- and T2-weighted sequences. The appearance of ONJ on X-rays and in the CT scan is variable and includes poorly defined areas with a permeated appearance showing cortical destruction, bone sequestration, periosteal reaction or sclerotic changes. The presence of sclerosis associated to a disorganized microtrabecular structure could represent the first imaging signs of BP-induced ONJ. The presence of a periosteal reaction and bone sequestration in turn is associated to more advanced stage disease (29).

Ruggiero et al. (30) have proposed a clinical classification that can be used for staging ONJ and for planning treatment. This system includes two new stages with respect to the previous classification: the designation of “risk category” for those patients treated with oral or intravenous bisphosphonates who do not show exposure of necrotic bone; and “stage 0” referred to those patients who in the absence of clinical evidence of bone exposure show nonspecific clinical manifestations such as mandibular pain or osteosclerosis (31). A new modification proposed by Bagán et al. (32), which has been supported by other authors (33), is described in  Table 3. In this classification stage 2 is subdivided according to whether the patient remains stable without progression or worsening of necrosis or of the signs derived from necrosis (stage 2a), or whether the condition progresses in terms of the extent of necrosis or of its derived infectious complications, without producing mandibular fracture, extraoral fistula or osteolysis (stage 2b). However, in contrast to the classification proposed by Ruggiero et al. (30) and posteriorly modified by Bagán et al. (32), Woo et al. (33) advises subdividing stage 0 according to the patient symptoms. Thus, a distinction is made between stage 0sa (suspected asymptomatic), corresponding to patients without bone exposure but with fistulas or deep periodontal pockets causing no symptoms and treated only with chlorhexidine rinses; and stage 0ss (suspected symptomatic), corresponding to patients without bone exposure but with fistulas or deep periodontal pockets causing symptoms and treated only with chlorhexidine rinses and systemic antibiotics.

**Table 3:**
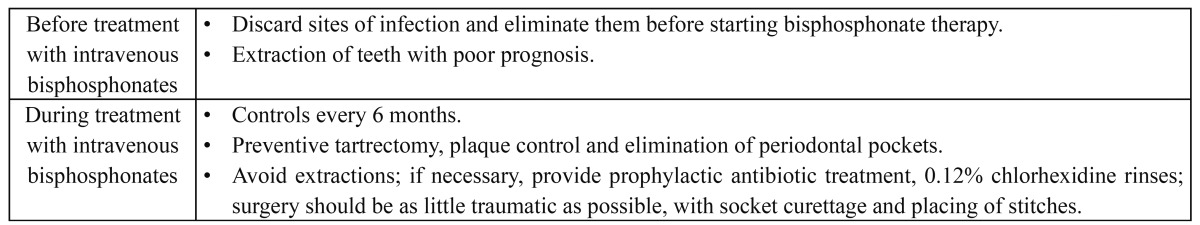
General recommendations in patients receiving intravenous bisphosphonate treatment (8).

The treatment of these patients remains the subject of debate. The objective of treatment in patients diagnosed with ONJ should be to eliminate the pain, control the soft tissue and bone infection, and avoid or reduce the progression of bone necrosis. Several authors (22,30) consider that the suppression of oral BP treatment for a period of 6-12 months results in clinical improvement and even spontaneous resolution of the condition. Suspension is therefore advisable, provided the systemic clinical conditions of the patient allow the interruption of BP therapy. Since 25% of trabecular bone and 3% of cortical bone are renewed each year, the interruption of BP treatment theoretically could have a beneficial effect, since the newly formed bone is unable to absorb BP (14). However, in a study involving 25 patients, BP suppression was not seen to exert an effect (9). Corticosteroid discontinuation also should be considered in patients concomitantly receiving these drugs as maintenance therapy (34). It has been suggested that these patients should receive conservative management, since the mucosal disruptions resolve in at least 23-53% of the cases after following a series of recommendations: administration of topical chlorhexidine and systemic antibiotics in cases of pain and infection, the suppression of BP treatment, or hyperbaric oxygen therapy (17,25). A preliminary study (27) of 10 patients has described a conservative treatment option based on direct ozone (O3) application in gel form, thereby facilitating ozone release over the necrotic bone. Beneficial results were obtained, since in two patients (20%) the radiological controls showed disappearance of the lesions and complete regeneration of the oral tissues. The shedding of bone sequestration was recorded in 8 patients (80%), with complete reepithelization of the lesions in two cases. There were no cases of ONJ relapse after 8 months of follow-up. Such therapy therefore should be regarded as an effective, safe and simple treatment option in application to BP-induced ONJ measuring ≤ 2.5 cm in size. Another described treatment option is the administration of isoprenoid geranyl diphosphate (metabolic form of geraniol), which reverts inhibition of the mevalonate pathway induced by nitrogenated bisphosphonates (35).

Bocanegra-Pérez et al. (36) recommend treating these patients on a conservative basis, administering oral antibiotics such as amoxicillin – clavulanic acid 1000/62.5 mg two tablets/day/30 days, or metronidazole 250 mg two tablets/8 hours/10-20 days, and 0.12% chlorhexidine rinses 3-4 times a day. The fistulas in turn can be treated with an intravenous perfusion of ciprofloxacin 2 mg/ml. In a case series published by Marx et al. (37), 90% of the patients in stages 1 and 2 were stabilized with conservative treatment in the form of oral rinses and systemic antibiotics. Another study (25) found 3-10% of the patients to fail to respond to conservative treatment or suffer pathological fractures – surgery being needed, with resection of the necrotic bone. The importance of aggressive treatment has been underscored in a study (38) in which conservative management was not effective. A surgical technique has been described, based on fluorescence guided bone resection of 20 ONJ zones in 15 patients – the success rate being 85% after four weeks of follow-up (12). The treatment response has been reported to be variable, with a poorer response in patients with maxillary sinusitis associated to ONJ (15). The treatment guidelines proposed by Bagán et al. (32) are described in  Table 2.

## Prevention

It has long been reported that the determination of CTX (C-terminal telopeptide of type 1 collagen) in the serum of patients treated with BP could be of use in predicting ONJ in patients subjected to oral surgery. Patients with CTX ≥150 pg/ml can undergo any type of surgery with only minimum risks and without the need to suspend the medication; however, in the presence of CTX < 150 pg/ml the risks increase (22). In contrast, other authors (39,40) have not found CTX to offer any true predictive capacity. As a result, its use must be viewed with caution, and the CTX values cannot be used as a definitive indicator of the risk of suffering BP-induced ONJ. Serum osteocalcin is another marker that could be of use in predicting the risk of ONJ. In this sense, concentrations below the normal limits could indicate problems with the bone formation process, and may be regarded as a risk factor (40). Periapical X-rays can also be important, revealing sclerotic areas and loss of the inferior alveolar nerve contour caused by progressive sclerosis (29).

It has been shown that the adoption of preventive measures before and during intravenous BP therapy in cancer patients with bone metastases and in individuals with multiple myeloma is accompanied by a 75% reduction in the incidence of ONJ. Whenever possible, such preventive measures should include adequate oral hygiene before administering BP treatment, together with the extraction of teeth showing a poor prognosis, caries control, and monitorization of the correct fitting of removable dentures (2).  Table 3 summarizes these recommendations, which moreover can be extrapolated to patients treated with bisphosphonates via the oral route (8).

All patients treated with bisphosphonates are at risk of developing osteonecrosis as a result of such medication. This potential complication therefore should be explained to the patient by both the prescribing physician and the dental surgeon in charge of oral treatment, with the obtainment of informed consent in all cases.
